# 1-[2-(2,5-Dichloro­benz­yloxy)-2-phenyl­eth­yl]-1*H*-benzotriazole

**DOI:** 10.1107/S1600536811044783

**Published:** 2011-11-05

**Authors:** Özden Özel Güven, Meral Bayraktar, Simon J. Coles, Tuncer Hökelek

**Affiliations:** a Department of Chemistry, Zonguldak Karaelmas University, 67100 Zonguldak, Turkey; b Department of Chemistry, Southampton University, Southampton SO17 1BJ, England; c Department of Physics, Hacettepe University, 06800 Beytepe, Ankara, Turkey

## Abstract

In the title mol­ecule, C_21_H_17_Cl_2_N_3_O, the benzotriazole ring is oriented at dihedral angles of 48.72 (6) and 62.94 (5)°, respectively, to the phenyl and benzene rings and the dihedral angle between the phenyl and benzene rings is 88.95 (6)°. In the crystal, weak C—H⋯N hydrogen bonds link the mol­ecules into chains. π–π contacts between the triazole and benzene rings [centroid–centroid distance = 3.678 (1) Å] and a weak C—H⋯π inter­action are also observed.

## Related literature

For general background to the biological activity of benzotriazole derivatives, see: Hirokawa *et al.* (1998 ▶); Yu *et al.* (2003 ▶); Kopanska *et al.* (2004 ▶). For related structures, see: Katritzky *et al.* (2001 ▶); Özel Güven *et al.* (2007*a* ▶,*b* ▶, 2010*a* ▶,*b* ▶, 2011 ▶); Swamy *et al.* (2006 ▶).
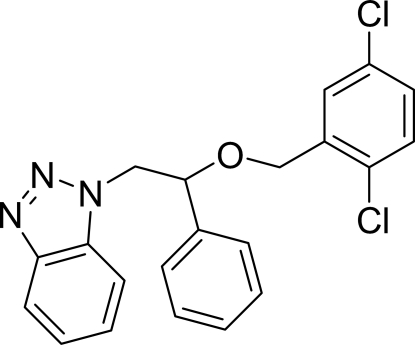


## Experimental

### 

#### Crystal data

C_21_H_17_Cl_2_N_3_O
*M* *_r_* = 398.28
Triclinic, 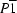

*a* = 8.6970 (3) Å
*b* = 8.8385 (3) Å
*c* = 13.3918 (4) Å
α = 105.840 (3)°
β = 104.453 (3)°
γ = 99.713 (2)°
*V* = 927.47 (6) Å^3^
*Z* = 2
Mo *K*α radiation
μ = 0.37 mm^−1^
*T* = 120 K
0.20 × 0.13 × 0.08 mm

#### Data collection

Bruker–Nonius KappaCCD diffractometer
Absorption correction: multi-scan (*SADABS*; Sheldrick, 2007 ▶) *T*_min_ = 0.930, *T*_max_ = 0.971
21356 measured reflections
4271 independent reflections
3391 reflections with *I* > 2σ(*I*)
*R*_int_ = 0.055

#### Refinement

*R*[*F* ^2^ > 2σ(*F* ^2^)] = 0.043
*wR*(*F* ^2^) = 0.120
*S* = 1.12
4271 reflections
244 parameters
H-atom parameters constrained
Δρ_max_ = 0.47 e Å^−3^
Δρ_min_ = −0.50 e Å^−3^

### 

Data collection: *COLLECT* (Nonius, 1998 ▶); cell refinement: *DENZO* (Otwinowski & Minor, 1997 ▶) and *COLLECT*; data reduction: *DENZO* and *COLLECT*; program(s) used to solve structure: *SHELXS97* (Sheldrick, 2008 ▶); program(s) used to refine structure: *SHELXL97* (Sheldrick, 2008 ▶); molecular graphics: *ORTEP-3 for Windows* (Farrugia, 1997 ▶); software used to prepare material for publication: *WinGX* (Farrugia, 1999 ▶) and *PLATON* (Spek, 2009 ▶).

## Supplementary Material

Crystal structure: contains datablock(s) I, global. DOI: 10.1107/S1600536811044783/su2333sup1.cif

Structure factors: contains datablock(s) I. DOI: 10.1107/S1600536811044783/su2333Isup2.hkl

Supplementary material file. DOI: 10.1107/S1600536811044783/su2333Isup3.cml

Additional supplementary materials:  crystallographic information; 3D view; checkCIF report

## Figures and Tables

**Table 1:** Hydrogen-bond geometry (Å, °) *Cg*1 is the centroid of the C2–C7 ring.

*D*—H⋯*A*	*D*—H	H⋯*A*	*D*⋯*A*	*D*—H⋯*A*
C19—H19⋯N3^i^	0.93	2.62	3.549 (2)	174
C18—H18⋯*Cg*1^ii^	0.93	2.69	3.605 (2)	168

Symmetry codes: (i) 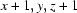; (ii) 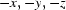.

**Fig. 1.**
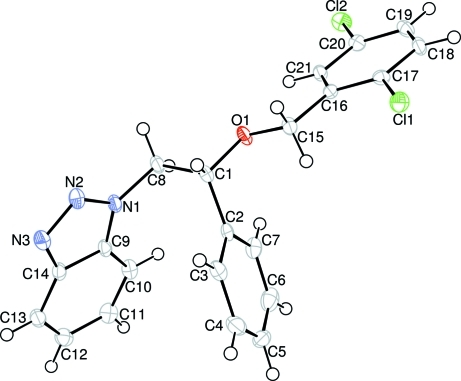
The molecular structure of the title compound with the atom-numbering scheme. Displacement ellipsoids are drawn at the 50% probability level.

**Fig. 2.**
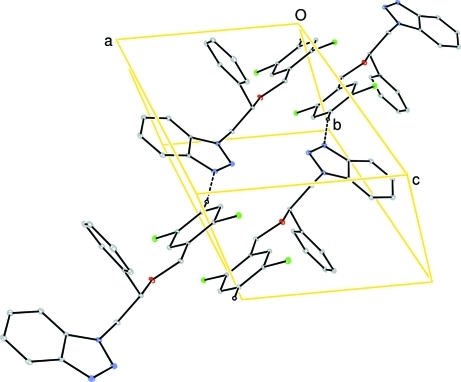
A partial packing diagram. Hydrogen bonds are shown as dashed lines. Hydrogen atoms not involved in hydrogen bonding have been omitted for clarity.

## supplementary crystallographic information

### Comment

Clotrimazole, miconazole, econazole, ketoconazole, itraconazole and fluconazole have been developed for clinical uses as antifungal agents. Lately, similar structures containing benzimidazole ring have been reported as antibacterial agents (Özel Güven *et al.*, 2007*a*,*b*). Benzotriazole derivatives also exhibit a good degree of analgesic, anti-inflammatory, diuretic, antiviral and antihypertensive activities (Hirokawa *et al.*, 1998; Yu *et al.*, 2003; Kopanska *et al.*, 2004). Crystal structures of benzotriazole ring possesing compounds have been reported before (Katritzky *et al.*, 2001; Swamy *et al.*, 2006; Özel Güven *et al.*, 2010*a*,*b*; Özel Güven *et al.*, 2011). Now, we report herein the crystal structure of the title benzotriazole derivative, (I).

In the molecule of the title compound (Fig. 1), the bond lengths and angles are generally within normal ranges. The planar benzotriazole ring [B (N1-N3/C9-C14)] is oriented with respect to the phenyl [A (C2-C7)] and benzene [C (C16-C21)] rings at dihedral angles of A/B = 48.72 (6) and B/C = 62.94 (5) °. The dihedral angle between phenyl and benzene rings is A/C = 88.95 (6)°. Atom C8 is -0.017 (2) Å away from the plane of the benzotriazole ring and atom C1 is 0.069 (2) Å away from the plane of the phenyl ring, while atoms O1 and C15 are -0.052 (1) and 0.010 (2) Å away from the plane of the benzene ring.

In the crystal, weak C—H···N hydrogen bonds (table 1) link the molecules into chains (Fig. 2). There also exists a π–π contact between the triazole and benzene rings, Cg4—Cg5^i^, may further stabilize the structure [centroid-centroid distance = 3.678 (1) Å; symmetry code: (i) 2 - x, 1 - y, 1 - z; Cg4 and Cg5 are the centroids of the rings D (N1—N3/C9/C14) and E (C9—C14)]. A weak C—H···π interaction (Table 1) may stabilize the structure.

### Experimental

The title compound was synthesized by the reaction of 2-(1*H*-benzotriazol-1-yl)-1-phenylethanol (Özel Güven *et al.*, 2010*a*) with aryl halide using NaH.2-(1*H*-benzotriazol-1-yl)-1- phenylethanol (200 mg, 0.84 mmol) was dissolved in DMF (3-4 ml). NaH (33 mg, 0.84 mmol) was added to the solution portionwise. After stirring the mixture a few minutes, 2,5-dichlorobenzyl bromide (200 mg, 0.84 mmol) was added dropwise. Then, the reaction mixture was stirred additional 3 h at room temperature. Adding methanol (5 ml) reaction was stopped. After evaporation of the solvent, dichloromethane was added to the reaction mixture and extracted with water. Then, the organic phase was separated, dried, filtered and evaporated. The precipitate formed was purified by column chromatography using chloroform and crystallized from *iso*-propanol to obtain colorless crystals suitable for X-ray analysis (yield; 231 mg, 69%).

### Refinement

H atoms were positioned geometrically with C—H = 0.98, 0.93 and 0.97 Å for methine, aromatic and methylene H, respectively, and constrained to ride on their parent atoms, with U_iso_(H) = 1.2U_eq_(C).

### Crystal data

**Table:**

C_21_H_17_Cl_2_N_3_O	*Z* = 2
*M**_r_* = 398.28	*F*(000) = 412
Triclinic, *P*1	*D*_x_ = 1.426 Mg m^−^^3^
Hall symbol: -P 1	Mo *K*α radiation, λ = 0.71073 Å
*a* = 8.6970 (3) Å	Cell parameters from 14295 reflections
*b* = 8.8385 (3) Å	θ = 2.9–27.5°
*c* = 13.3918 (4) Å	µ = 0.37 mm^−^^1^
α = 105.840 (3)°	*T* = 120 K
β = 104.453 (3)°	Block, colorless
γ = 99.713 (2)°	0.20 × 0.13 × 0.08 mm
*V* = 927.47 (6) Å^3^	

### Data collection

**Table:**

Bruker–Nonius KappaCCD diffractometer	4271 independent reflections
Radiation source: fine-focus sealed tube	3391 reflections with *I* > 2σ(*I*)
graphite	*R*_int_ = 0.055
φ and ω scans	θ_max_ = 27.6°, θ_min_ = 3.0°
Absorption correction: multi-scan (*SADABS*; Sheldrick, 2007)	*h* = −11→11
*T*_min_ = 0.930, *T*_max_ = 0.971	*k* = −11→11
21356 measured reflections	*l* = −17→16

### Refinement

**Table:**

Refinement on *F*^2^	Primary atom site location: structure-invariant direct methods
Least-squares matrix: full	Secondary atom site location: difference Fourier map
*R*[*F*^2^ > 2σ(*F*^2^)] = 0.043	Hydrogen site location: inferred from neighbouring sites
*wR*(*F*^2^) = 0.120	H-atom parameters constrained
*S* = 1.12	*w* = 1/[σ^2^(*F*_o_^2^) + (0.0596*P*)^2^ + 0.2097*P*] where *P* = (*F*_o_^2^ + 2*F*_c_^2^)/3
4271 reflections	(Δ/σ)_max_ = 0.001
244 parameters	Δρ_max_ = 0.47 e Å^−^^3^
0 restraints	Δρ_min_ = −0.50 e Å^−^^3^

### Special details

**Table:**

Geometry. All esds (except the esd in the dihedral angle between two l.s. planes) are estimated using the full covariance matrix. The cell esds are taken into account individually in the estimation of esds in distances, angles and torsion angles; correlations between esds in cell parameters are only used when they are defined by crystal symmetry. An approximate (isotropic) treatment of cell esds is used for estimating esds involving l.s. planes.
Refinement. Refinement of F^2^ against ALL reflections. The weighted R-factor wR and goodness of fit S are based on F^2^, conventional R-factors R are based on F, with F set to zero for negative F^2^. The threshold expression of F^2^ > 2sigma(F^2^) is used only for calculating R-factors(gt) etc. and is not relevant to the choice of reflections for refinement. R-factors based on F^2^ are statistically about twice as large as those based on F, and R- factors based on ALL data will be even larger.

### Fractional atomic coordinates and isotropic or equivalent isotropic displacement parameters (Å^2^)

**Table:**

	*x*	*y*	*z*	*U*_iso_*/*U*_eq_	
Cl1	1.14499 (5)	0.89780 (6)	0.93114 (4)	0.02094 (14)	
Cl2	0.71261 (6)	0.46919 (6)	1.10951 (4)	0.02373 (14)	
O1	0.61227 (15)	0.66913 (16)	0.77690 (10)	0.0196 (3)	
N1	0.25973 (19)	0.61345 (19)	0.54291 (12)	0.0178 (3)	
N2	0.2767 (2)	0.5667 (2)	0.44128 (12)	0.0224 (4)	
N3	0.1660 (2)	0.6091 (2)	0.37527 (12)	0.0229 (4)	
C1	0.5343 (2)	0.7104 (2)	0.68405 (14)	0.0164 (4)	
H1	0.5981	0.6965	0.6329	0.020*	
C2	0.5167 (2)	0.8836 (2)	0.71551 (14)	0.0171 (4)	
C3	0.5459 (2)	0.9801 (2)	0.65228 (15)	0.0224 (4)	
H3	0.5803	0.9394	0.5922	0.027*	
C4	0.5241 (3)	1.1372 (3)	0.67827 (18)	0.0297 (5)	
H4	0.5451	1.2016	0.6362	0.036*	
C5	0.4709 (2)	1.1970 (3)	0.76725 (18)	0.0307 (5)	
H5	0.4540	1.3009	0.7841	0.037*	
C6	0.4431 (2)	1.1023 (3)	0.83084 (17)	0.0304 (5)	
H6	0.4084	1.1432	0.8908	0.036*	
C7	0.4665 (2)	0.9469 (2)	0.80589 (15)	0.0230 (4)	
H7	0.4486	0.8844	0.8496	0.028*	
C8	0.3692 (2)	0.5830 (2)	0.63280 (14)	0.0200 (4)	
H8A	0.3884	0.4765	0.6063	0.024*	
H8B	0.3164	0.5821	0.6885	0.024*	
C9	0.1356 (2)	0.6899 (2)	0.54283 (14)	0.0172 (4)	
C10	0.0700 (2)	0.7613 (2)	0.62427 (15)	0.0214 (4)	
H10	0.1098	0.7626	0.6958	0.026*	
C11	−0.0566 (2)	0.8293 (3)	0.59197 (16)	0.0244 (4)	
H11	−0.1025	0.8795	0.6436	0.029*	
C12	−0.1196 (2)	0.8254 (2)	0.48260 (16)	0.0238 (4)	
H12	−0.2064	0.8717	0.4643	0.029*	
C13	−0.0554 (2)	0.7549 (2)	0.40323 (15)	0.0220 (4)	
H13	−0.0968	0.7527	0.3316	0.026*	
C14	0.0754 (2)	0.6861 (2)	0.43453 (14)	0.0179 (4)	
C15	0.7840 (2)	0.7424 (2)	0.81794 (14)	0.0184 (4)	
H15A	0.8037	0.8585	0.8309	0.022*	
H15B	0.8360	0.6971	0.7646	0.022*	
C16	0.8562 (2)	0.7120 (2)	0.92259 (14)	0.0157 (4)	
C17	1.0225 (2)	0.7779 (2)	0.98111 (14)	0.0159 (4)	
C18	1.0947 (2)	0.7531 (2)	1.07830 (14)	0.0190 (4)	
H18	1.2057	0.7998	1.1157	0.023*	
C19	1.0001 (2)	0.6582 (2)	1.11903 (14)	0.0197 (4)	
H19	1.0464	0.6388	1.1834	0.024*	
C20	0.8346 (2)	0.5930 (2)	1.06155 (14)	0.0176 (4)	
C21	0.7616 (2)	0.6186 (2)	0.96475 (14)	0.0173 (4)	
H21	0.6501	0.5735	0.9284	0.021*	

### Atomic displacement parameters (Å^2^)

**Table:**

	*U*^11^	*U*^22^	*U*^33^	*U*^12^	*U*^13^	*U*^23^
Cl1	0.0188 (2)	0.0214 (3)	0.0219 (2)	0.00319 (19)	0.00663 (19)	0.00705 (19)
Cl2	0.0264 (3)	0.0283 (3)	0.0226 (2)	0.0087 (2)	0.0116 (2)	0.0132 (2)
O1	0.0155 (7)	0.0245 (7)	0.0166 (6)	0.0027 (5)	−0.0012 (5)	0.0103 (5)
N1	0.0169 (8)	0.0191 (8)	0.0136 (7)	0.0020 (6)	0.0000 (6)	0.0053 (6)
N2	0.0253 (9)	0.0251 (9)	0.0137 (7)	0.0057 (7)	0.0029 (7)	0.0050 (7)
N3	0.0253 (9)	0.0257 (9)	0.0157 (7)	0.0075 (7)	0.0026 (7)	0.0066 (7)
C1	0.0153 (9)	0.0209 (9)	0.0124 (8)	0.0048 (7)	0.0015 (7)	0.0070 (7)
C2	0.0088 (8)	0.0200 (9)	0.0168 (8)	0.0013 (7)	−0.0019 (7)	0.0042 (7)
C3	0.0172 (10)	0.0272 (11)	0.0205 (9)	0.0043 (8)	0.0015 (8)	0.0094 (8)
C4	0.0226 (11)	0.0273 (11)	0.0355 (12)	0.0043 (9)	−0.0021 (9)	0.0160 (9)
C5	0.0179 (10)	0.0170 (10)	0.0434 (12)	0.0060 (8)	−0.0049 (9)	0.0007 (9)
C6	0.0184 (10)	0.0312 (12)	0.0318 (11)	0.0041 (9)	0.0057 (9)	−0.0014 (9)
C7	0.0178 (10)	0.0261 (11)	0.0200 (9)	0.0016 (8)	0.0044 (8)	0.0033 (8)
C8	0.0198 (10)	0.0202 (10)	0.0157 (8)	0.0025 (8)	−0.0020 (7)	0.0074 (7)
C9	0.0144 (9)	0.0175 (9)	0.0155 (8)	−0.0013 (7)	−0.0001 (7)	0.0065 (7)
C10	0.0186 (10)	0.0256 (10)	0.0177 (9)	0.0013 (8)	0.0046 (8)	0.0074 (8)
C11	0.0214 (10)	0.0270 (11)	0.0240 (10)	0.0037 (8)	0.0086 (8)	0.0072 (8)
C12	0.0165 (10)	0.0230 (10)	0.0291 (10)	0.0025 (8)	0.0026 (8)	0.0101 (8)
C13	0.0218 (10)	0.0204 (10)	0.0194 (9)	0.0005 (8)	0.0000 (8)	0.0086 (8)
C14	0.0180 (9)	0.0166 (9)	0.0143 (8)	−0.0011 (7)	0.0005 (7)	0.0049 (7)
C15	0.0169 (9)	0.0193 (10)	0.0159 (8)	0.0033 (8)	0.0010 (7)	0.0055 (7)
C16	0.0169 (9)	0.0148 (9)	0.0132 (8)	0.0068 (7)	0.0029 (7)	0.0013 (7)
C17	0.0157 (9)	0.0156 (9)	0.0184 (8)	0.0064 (7)	0.0070 (7)	0.0056 (7)
C18	0.0132 (9)	0.0241 (10)	0.0155 (8)	0.0065 (8)	0.0003 (7)	0.0024 (7)
C19	0.0210 (10)	0.0270 (10)	0.0135 (8)	0.0124 (8)	0.0050 (7)	0.0072 (8)
C20	0.0188 (9)	0.0200 (9)	0.0162 (8)	0.0069 (8)	0.0079 (7)	0.0060 (7)
C21	0.0142 (9)	0.0188 (9)	0.0158 (8)	0.0052 (7)	0.0018 (7)	0.0032 (7)

### Geometric parameters (Å, °)

**Table:**

Cl1—C17	1.7518 (19)	C8—H8B	0.9700
Cl2—C20	1.7514 (19)	C9—C10	1.404 (3)
O1—C1	1.432 (2)	C10—C11	1.374 (3)
O1—C15	1.422 (2)	C10—H10	0.9300
N1—N2	1.363 (2)	C11—H11	0.9300
N1—C8	1.456 (2)	C12—C11	1.418 (3)
N1—C9	1.366 (2)	C12—H12	0.9300
N3—N2	1.314 (2)	C13—C12	1.372 (3)
N3—C14	1.383 (2)	C13—C14	1.405 (3)
C1—C8	1.530 (3)	C13—H13	0.9300
C1—H1	0.9800	C14—C9	1.403 (2)
C2—C1	1.517 (3)	C15—H15A	0.9700
C2—C3	1.392 (3)	C15—H15B	0.9700
C2—C7	1.395 (3)	C16—C15	1.501 (2)
C3—C4	1.395 (3)	C16—C17	1.395 (3)
C3—H3	0.9300	C18—C17	1.390 (3)
C4—H4	0.9300	C18—H18	0.9300
C5—C4	1.388 (3)	C19—C18	1.385 (3)
C5—H5	0.9300	C19—C20	1.386 (3)
C6—C5	1.381 (3)	C19—H19	0.9300
C6—H6	0.9300	C21—C16	1.391 (3)
C7—C6	1.385 (3)	C21—C20	1.390 (2)
C7—H7	0.9300	C21—H21	0.9300
C8—H8A	0.9700		
			
C15—O1—C1	111.82 (13)	C9—C10—H10	122.0
N2—N1—C8	120.18 (15)	C11—C10—C9	115.98 (17)
N2—N1—C9	110.27 (14)	C11—C10—H10	122.0
C9—N1—C8	129.53 (15)	C10—C11—C12	122.25 (19)
N3—N2—N1	108.69 (15)	C10—C11—H11	118.9
N2—N3—C14	108.37 (15)	C12—C11—H11	118.9
O1—C1—C2	112.30 (14)	C11—C12—H12	119.2
O1—C1—C8	103.20 (14)	C13—C12—C11	121.54 (19)
O1—C1—H1	109.3	C13—C12—H12	119.2
C2—C1—C8	113.11 (15)	C12—C13—C14	117.29 (17)
C2—C1—H1	109.3	C12—C13—H13	121.4
C8—C1—H1	109.3	C14—C13—H13	121.4
C3—C2—C1	120.21 (16)	N3—C14—C9	108.34 (16)
C3—C2—C7	119.01 (18)	N3—C14—C13	131.14 (17)
C7—C2—C1	120.75 (16)	C9—C14—C13	120.51 (17)
C2—C3—C4	120.57 (19)	O1—C15—C16	109.65 (14)
C2—C3—H3	119.7	O1—C15—H15A	109.7
C4—C3—H3	119.7	O1—C15—H15B	109.7
C3—C4—H4	120.2	C16—C15—H15A	109.7
C5—C4—C3	119.61 (19)	C16—C15—H15B	109.7
C5—C4—H4	120.2	H15A—C15—H15B	108.2
C4—C5—H5	120.0	C17—C16—C15	120.42 (16)
C6—C5—C4	120.05 (19)	C21—C16—C15	121.89 (16)
C6—C5—H5	120.0	C21—C16—C17	117.70 (16)
C5—C6—C7	120.41 (19)	C16—C17—Cl1	118.97 (14)
C5—C6—H6	119.8	C18—C17—Cl1	118.58 (14)
C7—C6—H6	119.8	C18—C17—C16	122.45 (17)
C2—C7—H7	119.8	C17—C18—H18	120.3
C6—C7—C2	120.33 (19)	C19—C18—C17	119.48 (17)
C6—C7—H7	119.8	C19—C18—H18	120.3
N1—C8—C1	112.73 (15)	C18—C19—C20	118.36 (16)
N1—C8—H8A	109.0	C18—C19—H19	120.8
N1—C8—H8B	109.0	C20—C19—H19	120.8
C1—C8—H8A	109.0	C19—C20—Cl2	119.43 (14)
C1—C8—H8B	109.0	C19—C20—C21	122.33 (17)
H8A—C8—H8B	107.8	C21—C20—Cl2	118.22 (14)
N1—C9—C10	133.25 (16)	C16—C21—H21	120.2
N1—C9—C14	104.33 (16)	C20—C21—C16	119.67 (17)
C14—C9—C10	122.42 (18)	C20—C21—H21	120.2
			
C15—O1—C1—C2	−74.67 (18)	C2—C7—C6—C5	−0.7 (3)
C15—O1—C1—C8	163.19 (14)	N1—C9—C10—C11	179.31 (19)
C1—O1—C15—C16	173.24 (14)	C14—C9—C10—C11	−0.6 (3)
C8—N1—N2—N3	−179.18 (16)	C9—C10—C11—C12	1.1 (3)
C9—N1—N2—N3	−0.7 (2)	C13—C12—C11—C10	−0.9 (3)
N2—N1—C8—C1	81.4 (2)	C14—C13—C12—C11	0.1 (3)
C9—N1—C8—C1	−96.7 (2)	C12—C13—C14—N3	−179.28 (19)
N2—N1—C9—C10	−179.3 (2)	C12—C13—C14—C9	0.4 (3)
N2—N1—C9—C14	0.61 (19)	N3—C14—C9—N1	−0.3 (2)
C8—N1—C9—C10	−1.0 (3)	N3—C14—C9—C10	179.59 (17)
C8—N1—C9—C14	178.92 (17)	C13—C14—C9—N1	179.96 (17)
C14—N3—N2—N1	0.5 (2)	C13—C14—C9—C10	−0.1 (3)
N2—N3—C14—C9	−0.1 (2)	C17—C16—C15—O1	−177.33 (15)
N2—N3—C14—C13	179.59 (19)	C21—C16—C15—O1	2.6 (2)
O1—C1—C8—N1	173.19 (14)	C15—C16—C17—Cl1	0.5 (2)
C2—C1—C8—N1	51.6 (2)	C15—C16—C17—C18	179.99 (17)
C3—C2—C1—O1	139.00 (17)	C21—C16—C17—Cl1	−179.40 (13)
C3—C2—C1—C8	−104.68 (19)	C21—C16—C17—C18	0.1 (3)
C7—C2—C1—O1	−43.0 (2)	C19—C18—C17—Cl1	−179.81 (14)
C7—C2—C1—C8	73.4 (2)	C19—C18—C17—C16	0.7 (3)
C1—C2—C3—C4	177.58 (17)	C20—C19—C18—C17	−1.0 (3)
C7—C2—C3—C4	−0.5 (3)	C18—C19—C20—Cl2	178.73 (14)
C1—C2—C7—C6	−176.83 (17)	C18—C19—C20—C21	0.5 (3)
C3—C2—C7—C6	1.2 (3)	C20—C21—C16—C15	179.49 (16)
C2—C3—C4—C5	−0.8 (3)	C20—C21—C16—C17	−0.6 (3)
C6—C5—C4—C3	1.3 (3)	C16—C21—C20—Cl2	−177.96 (13)
C7—C6—C5—C4	−0.6 (3)	C16—C21—C20—C19	0.3 (3)

### Hydrogen-bond geometry (Å, °)

**Table:**

Cg1 is the centroid of the C2–C7 ring.

**Table:**

*D*—H···*A*	*D*—H	H···*A*	*D*···*A*	*D*—H···*A*
C19—H19···N3^i^	0.93	2.62	3.549 (2)	174
C18—H18···Cg1^ii^	0.93	2.69	3.605 (2)	168

Symmetry codes: (i) *x*+1, *y*, *z*+1; (ii) −*x*, −*y*, −*z*.
